# 

**Published:** 1883-03

**Authors:** 


### BOOKS AND PAMPHLETS RECEIVED.

Legal Medicine. Tidy. Philadelphia: H. C. Lea's Son & Co., Jansen McClurg & Co.

Ohio Medical Society Transactions, 36th and 37th annual meetings.

Practical Examination of Urine. Tyson. P. Blakiston, Son & Co.

A Dictionary of Medicine. Quain. New York: D. Appleton & Co

Legal Medicine. Tidy. 2 vols. New York: Wm. Wood & Co. W. T. Keener.

Pocket Therapeutics and Dose Book. Stewart. Detroit: Stewart & Co.

Compend of Anatomy. Roberts. Philadelphia: C. C. Roberts & Co.

Manual of Gynecology. Hart & Barbour. New York: Wm. Wood & Co. W. T. Keener, Chicago.

Treatise on Fractures. Stimson. Philadelphia: Henry C. Lea's Son & Co. Jansen, McClurg & Co.

Diseases of the Ear. Politzer. Philadelphia: H. C. Lea's Son & Co. 1883. Jansen, McClurg & Co.

Early Aid in Injuries and Accidents. Esmarch. Philadelphia: H. C. Lea's Son & Co. Jansen, McClurg & Co.

Treatise on Diseases of the Skin. Hyde. Philadelphia: H. C. Lea's Son & Co. Jansen McClurg & Co.

Diseases of the Liver. Harley. Philadelphia: P. Blakiston, Son & Co. W. T. Keener.

---

Dr. R. G. Bogue has been appointed by the Cook County Commissioners one of the surgeons to Cook County Hospital. This selection is highly creditable to the Board, and if Dr. Bogue accepts, the medical students and the patients will have an able and conscientious practitioner and an eminently practical teacher.

Dr. E. S. Pettijohn, of Chicago, has been appointed second physician to the Eastern Hospital for Insane at Kankakee, to fill the place made vacant temporarily by absence of Dr. H. M. Mayer.

CLINICS.

MONDAY.

Eye and Ear Infirmary—1.15 to 2.15 p. m., Otological, by Dr. Schaefer; 2.15 to 3.30 p. m., Ophthalmological, Dr. Holmes.
Mercy Hospital—2 p. m., Medical, Profs. Hollister and Quine.
Rush Medical College—2 p. m., Medical by Prof. Bridge; 3 p. m., Dermatological and Venereal, by Prof. Hyde.
Woman's Medical College—2 p. m., Dermatological and Venereal, by Prof. Maynard.

TUESDAY.

Cook Co. Hospital—2 to 4 p. m., Medical and Surgical Clinics.
Mercy Hospital—2 p. m., Surgical Clinic, by Prof. Andrews.
Woman's Medical College—10 a. m., Prof. Ingals.

WEDNESDAY.

Chicago Medical College—2 p. m., Eye and Ear, by Prof. Jones.
Rush Medical College—3 p. m., Ophthalmological and Otological, by Prof. Holmes: 3 to 4 p. m., Diseases of the Chest, by Prof. Ross.
Woman's Medical College—2 p. m., Eye and Ear, by Dr. W. T. Montgomery; 3 p. m., Diseases of Children, by Prof. Chas. W. Earle.
Eye and Ear Infirmary—2.30 p. m., Dr. E. J. Gardiner.

THURSDAY.

Chicago Medical College—2 p. m., Gynæcological, Prof. Dudley.
Rush Medical College—2 p. m., Diseases of Children, by Prof. Knox; 3 p. m., Diseases of the Nervous System, by Prof. Lyman.
Eye and Ear Infirmary—1.15 to 2.25 p. m., Otological, by Dr. Schaefer; 2.15 to 3.30, p. m., Ophthalmological, Dr. Holmes.
Woman's Medical College—3 p. m., Surgical, by Prof. Owens.
College of Physicians and Surgeons—2 p. m., Medical, by Prof. S. A. McWilliams; 3 p. m., Surgical, by Prof. R. L. Rea.

FRIDAY.

Cook County Hospital—2 to 4 p. m., Medical and Surgical Clinics.
Mercy Hospital—2 p. m., Medical, by Prof. Davis.

SATURDAY.

Rush Medical College—2 p. m., Surgical, by Prof. Gunn.
Mercy Hospital—2 p. m., Surgical Clinic, by Prof. Andrews.
Chicago Medical College—3 p. m., Neurological, Prof. Jewell.
Woman's Medical College—2 p. m., Gynæcological, by Prof. T. D. Fitch.
College of Physicians and Surgeons—2 p. m., Diseases of the Chest, by Prof. S. A. McWilliams; 3 p. m., Gynæcological, by Prof. A. Reeves Jackson.

Daily Clinics, from 2 to 4 p. m., at the Central Free Dispensary, at the South Side Dispensary and at the West Side Dispensary.

